# Effectiveness of a Novel Covered Stent without External Thread Fixation for Anastomotic Leakage after Total or Proximal Gastrectomy for Gastric Cancer

**DOI:** 10.3390/cancers13153720

**Published:** 2021-07-23

**Authors:** Young-Il Kim, Chan Gyoo Kim, Jong Yeul Lee, Il Ju Choi, Bang Wool Eom, Hong Man Yoon, Keun Won Ryu, Young-Woo Kim

**Affiliations:** Center for Gastric Cancer, National Cancer Center, 323 Ilsan-ro, Goyang 10408, Korea; 11996@ncc.re.kr (Y.-I.K.); jylee@ncc.re.kr (J.Y.L.); cij1224@ncc.re.kr (I.J.C.); kneeling79@ncc.re.kr (B.W.E.); red10000@ncc.re.kr (H.M.Y.); docryu@ncc.re.kr (K.W.R.); gskim@ncc.re.kr (Y.-W.K.)

**Keywords:** Beta stent, anastomotic leakage, gastrectomy, gastric cancer

## Abstract

**Simple Summary:**

A covered self-expandable metal stent using external fixation using silk thread (thread-fix stent) is an effective treatment for anastomotic leakage after esophago-gastric surgery. However, a thread-fix stent also entails long hospitalization and patient discomfort. This study found that the Niti-S Beta stent which does not need thread-fix was an effective treatment for anastomotic leakage after total or proximal gastrectomy for gastric cancer. Because patients who received the Nitis-S Beta stent had minimal discomfort, the stent maintenance was possible without hospitalization.

**Abstract:**

A thread-fix stent entails long hospitalization and patient discomfort. We aimed to evaluate the efficacy of a novel stent with silicone-covered outer double layers without external fixation (Beta stent) for anastomotic leakage after total or proximal gastrectomy. The outcomes were compared between gastric cancer patients who underwent stent placement using a thread-fix stent between 2014 and 2015 (Thread-Fix Group) and those who received a Beta stent in the succeeding period until October 2018 (Beta Stent Group). The Beta Stent Group (*n* = 14) had a significantly higher leakage healing rate by the first stent placement (92.9% vs. 53.8%; *p* = 0.021) and had a shorter hospitalization period (median: 16 days vs. 28 days; *p* = 0.037) than the Thread-Fix Group (*n* = 13). Further, 50% of the Beta stent patients received outpatient management until stent removal. Stent maintenance duration was significantly longer in the Beta Stent Group (median, 28 days vs. 18 days; *p* = 0.006). There was no significant between-group difference in stent-related complications except for stent migration (7.1% (Beta Stent Group) vs. 0% (Thread-Fix Group), *p* = 0.326). In conclusion, the Niti-S Beta stent is an effective treatment for anastomotic leakage from total or proximal gastrectomy for gastric cancer. Stent maintenance is possible without hospitalization.

## 1. Introduction

Anastomotic leakage is one of the most serious and potentially fatal complications after gastrectomy, impairing quality of life, prolonging hospitalization, and increasing mortality [1,2,3]. Depending on the reconstruction method, anastomotic leakage occurs in 0–17% of patients after total gastrectomy [4] and in 0–5% after proximal gastrectomy [5,6,7]. The therapeutic options for anastomotic leakage after total or proximal gastrectomy include conservative, endoscopic, and surgical treatments. Among these, surgery is associated with extremely high mortality rates of up to 20–64% [8,9,10] and is thus only considered in select patients with severe symptoms and conditions such as sepsis with organ failure, diffuse peritonitis, and jejunal loop ischemia [11].

Meanwhile, conservative treatment involving intravenous antibiotics, fasting, and nutritional supports are helpful only in clinically stable patients with small leakage [11]. Additional endoscopic treatments are needed to improve leakage healing in patients with large anastomotic leakage. Among endoscopic modalities, fully covered self-expanding metallic stents (SEMSs) are highly effective for esophageal anastomotic leakage after gastrectomy, with treatment success rates of 70–85.7% [12,13,14]. However, stent migration can occur in up to 61% of patients who underwent esophagojejunostomy [12], and thus, external fixation through the nose of SEMS using a silk thread (thread-fix stent) was developed. This successfully eliminated the risk of stent migration [14,15]. Nonetheless, the thread-fix stent is associated with prolonged discomfort and longer hospitalization than the SEMS without external fixation due to the external fixation state during leakage healing.

Two recent studies reported that a newly designed fully covered SEMS with outer double layers (Niti-S Beta stent) is effective for leakage that develops after bariatric surgery, with low rate of stent migration (0–32%) [16,17]. The present study aimed to investigate the efficacy of the Niti-S Beta stent for the treatment of anastomotic leakage from total or proximal gastrectomy for gastric cancer. Towards this goal, we compared the rates of leakage healing rate and stent migration between the thread-fix stent and the Niti-S Beta stent.

## 2. Materials and Methods

### 2.1. Study Design and Patients

This was a retrospective study of gastric cancer patients who underwent gastrectomy between January 2014 and October 2018 at the National Cancer Center, Korea. Among them, patients who received fully covered SEMSs for anastomotic leakage after total or proximal gastrectomy were eligible. Patients who were treated only conservatively or received only other endoscopic procedures such as endoscopic clips with/without snares were excluded. The data of baseline clinical characteristics, anastomotic leakage characteristics and management, and treatment outcomes were collected.

### 2.2. Gastrectomy Protocol

All patients underwent total or proximal gastrectomy with lymph node dissection, by experienced surgeons. The reconstruction methods were Roux-en-Y esophago-jejunostomy for total gastrectomy and esophago-gastrostomy or double tract reconstruction for proximal gastrectomy. D1+ or D2 lymph node dissection was performed following the Japanese gastric cancer treatment guideline [18].

### 2.3. Diagnosis and Initial Management of Anastomotic Leakage

Post-gastrectomy computed tomography or endoscopy was performed in patients who had clinical presentations of anastomotic leakage such as signs of peritonitis, digestive fluids or abnormal fluid staining in the surgical drain, and unexplained elevation of white blood cell count or C-reactive protein. Initial management for anastomotic leakage included fasting with nutritional support, intravenous antibiotics, and percutaneous drainage of abnormal fluid collection around the leakage site.

### 2.4. Stent Placement and Follow-Up

**Figure 1:**
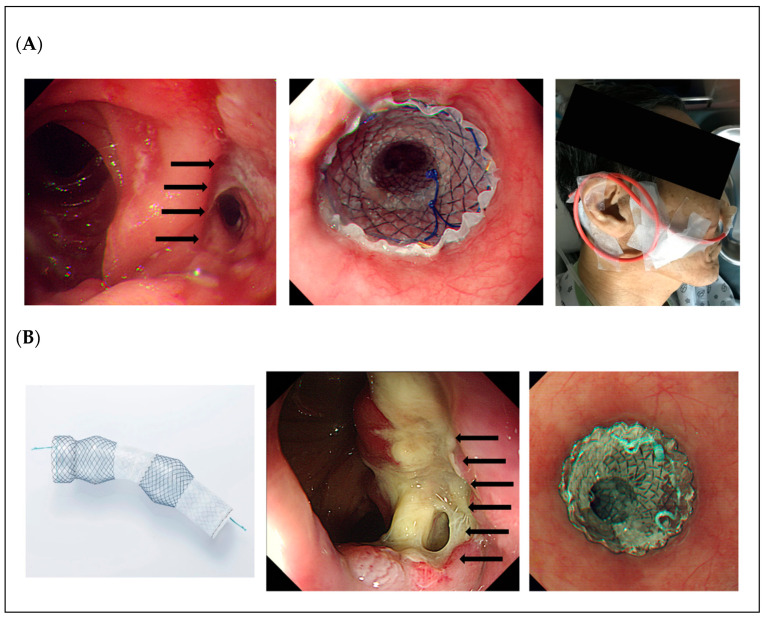
Stent placement for anastomotic leakage after gastrectomy. (**A**) Thread-fix stent placement is performed for anastomotic leakage after total gastrectomy and externally fixed through the nose using silk thread. (**B**) Niti-S Beta stent has a unique body structure with silicone-covered outer double layers, and the stent is placed for anastomotic leakage after total gastrectomy without external fixation.

Two types of fully covered stent for anastomotic leakage were used. The thread-fix stent (CHOOSTENT^TM^, M.I.Tech, Gyeong-gi, Korea) was used between January 2014 and December 2015 (Thread-Fix Stent Group). After introduction of the Niti-S Beta stent (BETA^TM^ Esophageal stent, Taewoong Medical, Gyeong-gi, Korea), we used the Beta stent between January 2016 and October 2018 (Beta Stent Group). Endoscopic stent placement was performed under fluoroscopic guidance. The guidewire was inserted through the endoscope, and a stent was deployed across the anastomotic leakage level. For patients who underwent the thread-fix SEMS, the stent thread was fixed through the nose with a similar technique used in an endoscopic nasobiliary drainage (ENBD) procedure (Figure 1A). Meanwhile, patients who underwent Niti-S Beta stent did not require external fixation because of the newly designed structure with silicone-covered outer double layers to prevent stent migration (Figure 1B). 

Contrast leakage was determined using upper gastrointestinal series (UGIS) imaging one day after stent placement. Follow-up endoscopy examinations were performed to identify the stent position and stent-related mucosal changes (erosion or ulcer) at day 2 post-placement then weekly or biweekly until stent removal. In the Thread-Fix Stent Group, improvements in anastomotic leakage status were assessed during follow-up endoscopy after stent reposition. Oral feeding was started from sips of water to a normal diet was initiated according to the patients’ clinical conditions. Patients who had stable clinical conditions after starting the diet were discharged and managed at the outpatient clinic.

### 2.5. Outcome Measures

The primary outcome measure was the success rate of complete leakage healing by the first stent placement without additional endoscopic procedures. The secondary outcome measures were the interval between stent placement and initiation of diet, duration of hospitalization in days, the proportion of patients discharged with stent maintenance status, the total duration from stent insertion to leakage closure, and stent placement–related complications. The duration of hospitalization was defined as the interval between the date of stent placement and of discharge.

### 2.6. Statistical Analysis

Continuous variables were presented as the median and interquartile range (IQR), and categorical variables were shown as proportions (%). Between-group differences were performed using the Mann–Whitney *U* test for continuous variables and the Chi-square test or Fisher’s exact test for categorical variables. All statistical analyses were performed using STATA version 13.1 (StataCorp, College Station, TX, USA), and *p*-values of less than 0.05 were considered statistically significant.

## 3. Results

### 3.1. Patient Characteristics

**Figure 2:**
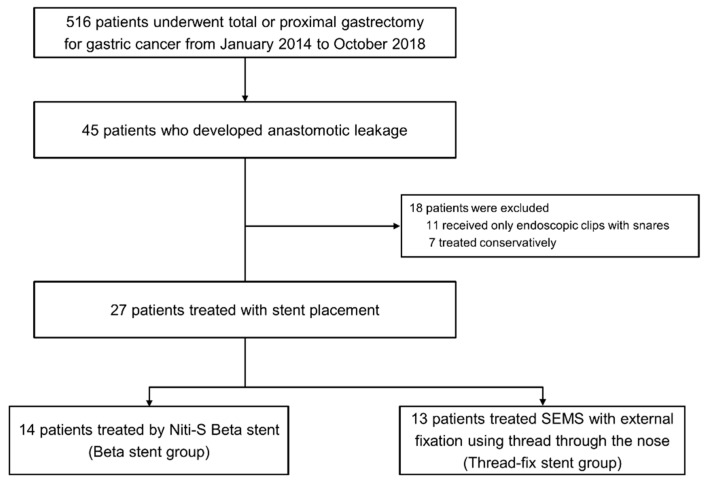
Patient inclusion flowchart.

Among the 2602 patients identified, 516 patients underwent total (*n* = 420 patients) or proximal gastrectomy (*n* = 96 patients). Of them, 45 patients who underwent total gastrectomy (37/420, 8.8%) and proximal gastrectomy (8/96, 8.3%) developed anastomotic leakage. After excluding 7 patients who have received only conservative treatment and 11 patients who received only endoscopic clips with or without snares without stent placement, 27 patients who underwent anastomotic leakage treatment with stent placement were included in the present study. There were 14 and 13 patients who underwent Niti-S Beta stent placement and thread-fix stent placement, respectively (Figure 2).

The baseline patient characteristics are shown in Table 1. The median patient age was 66 years, and majority (96.3%) were male. There was no significant between-group difference in the baseline clinical characteristics including age, sex, body mass index, ASA score, gastric cancer type, type of surgery, and pathological tumor stage.

### 3.2. Characteristics and Initial Management for Anastomotic Leakage

Table 2 shows the characteristics and initial management before stent placement. The median interval between gastrectomy and leakage diagnosis was 8 days in the Beta Stent Group and 7 days in the Thread-Fix Stent Group. The proportion of patients who underwent total gastrectomy was a significantly higher in the Thread-Fix Stent Group than in the Beta Stent Group (100% vs. 71.4%, respectively; *p* = 0.037). However, the distribution of the anastomotic leakage site, leakage size, and interval between leakage diagnosis and stent placement were not significantly different between the two groups. Before stent placement, percutaneous drainage was performed in 50.0% (7/14 patients) of the patients in the Beta Stent Group and in 53.8% (7/13 patients) in the Thread-Fix Stent Group. In addition, 3 patients (21.4%) in the Beta Stent Group and 2 patients (15.4%) in the Thread-Fix Stent Group underwent an endoscopic procedure (e.g., clips with or without detachable snares) before the stent placement.

### 3.3. Treatment Outcomes After Stent Placement

**Figure 3:**
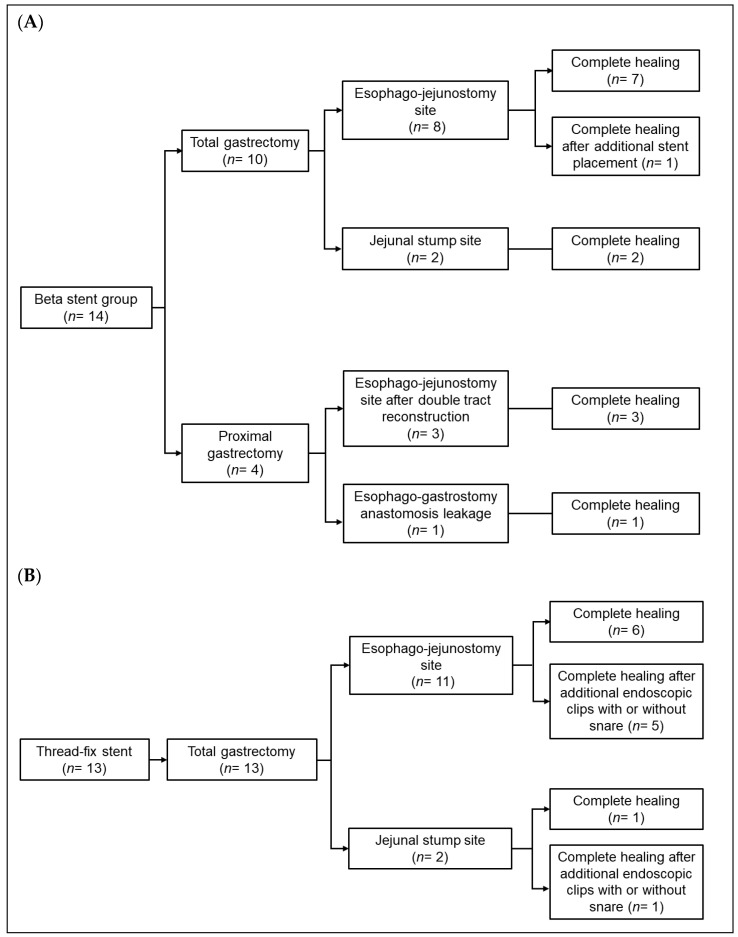
Detailed treatment outcomes after stent placement. (**A**) Beta Stent Group. (**B**) Thread-Fix Group.

The anastomotic leakage sites in the Beta Stent Group were the esophago-jejunostomy site in 11 patients (total gastrectomy, 8 patients; proximal gastrectomy, 3 patients), esophago-gastrostomy site after proximal gastrectomy in 1 patient, and blind jejunal pouch after total gastrectomy in 2 patients. Until complete anastomotic leakage healing, only one stent procedure was performed in 13 patients (92.9%). The remaining patient received an additional Niti-S Beta stent with a larger diameter (24 mm) after removal of the previous stent (20 mm diameter) because of persistent contrast leakage on UGIS imaging (Figure 3A).

Meanwhile, the anastomotic leakage sites in the Thread-Fix Stent Group were the esophago-jejunostomy site in 11 patients and blind jejunal pouch in 2 patients. To achieve complete leakage healing, only one stent procedure was performed in 7 patients (53.8%), and additional endoscopic procedures using clips with or without detachable snares were required in 6 patients (46.2%) (Figure 3B). Successful complete leakage healing was achieved in all patients. However, the rate of complete leakage healing in the first stent placement was significantly higher in the Niti-S Beta Stent Group than in the Thread-Fix Stent Group (92.9% (13/14) vs. 53.8% (7/13), *p* = 0.021).

The other treatment outcomes and complications related to the stent insertion are described in Table 3. Although there was no significant difference, the interval from stent placement to soft diet initiation was shorter in the Beta Stent Group (median, 6 days vs. 14 days, *p* = 0.118). Compared with the Thread-Fix Stent Group, the Beta Stent Group had a significantly shorter duration of hospitalization (median, 16 days vs. 32 days; *p* = 0.037) and significantly higher proportion of patients discharged with stent maintenance status (50.0% vs. 0%, *p* = 0.003). The duration of stent maintenance was also significantly longer in the Beta Stent Group (median, 28 days vs. 18 days, *p* = 0.006).

However, there were no significant between-group differences with respect to the time interval between leakage diagnosis and complete leakage healing and to the proportions of stent placement-related complications (e.g., stent site erosion and reflux symptoms). Stent migration occurred only in one patient in the Beta Stent Group. This patient developed esophago-gastrostomy anastomosis site leakage after proximal gastrectomy, and the migrated stent was repositioned to the leakage site using an endoscopic grasping forceps. Stent site stricture occurred in 3 patients in the Thread-Fix Stent Group, and the stricture was relieved via endoscopic balloon dilatations in all patients.

Until December 2020, long-term stent related complications were not developed. However, cancer recurrence occurred in a total of 3 patients (2 in the Thread-Fix Stent Group and one in the Beta Stent Group). All the patients received a systemic chemotherapy for peritoneal cancer recurrence, but died due to the cancer recurrence (survival months after the complete leakage closure: 12 months~28 months).

## 4. Discussion

Endoscopic treatment using SEMSs is an important treatment option for anastomotic leakage after esophago-gastric surgery for cancer [11]. However, the thread-fix stent used to prevent stent migration is also associated with prolonged discomfort and longer hospitalization. The present study found that compared with a thread-fix stent, the Niti-S Beta stent achieves a significantly higher success rate of complete leakage healing by first stent placement without additional endoscopic procedures. In addition, the Beta stent achieved a significantly longer stent maintenance duration without delay of leakage healing, and half of the patients in the Beta Stent Group received outpatient management until stent removal. Meanwhile, there was no significant difference in complications including stent migration between the two stents. To our best knowledge, the present study is the first to evaluate the effectiveness of the novel Niti-S Beta stent for the treatment of anastomotic leakage after total or proximal gastrectomy for gastric cancer.

The Niti-S Beta stent is a novel stent designed to minimize the risk of stent migration and to simplify the removal of stents for leakage or fistula after bariatric surgery. Two recent studies showed that the Niti-S Beta stent is an effective endoscopic treatment for leakage developing after bariatric surgery [16,17]. Tringali et al. reported a high rate of successful leakage healing by the first stent placement (80%, 8/10 patients) [16]. Boerlage et al. also reported a 100% success rate of leakage healing in patients who received the Niti-S Beta stent as initial treatment [17]. A case report also showed that duodenal leakage after right hemicolectomy was successfully treated using the Niti-S Beta stent [19]. In the current study, the Niti-S Beta stent was highly effective, with leakage healing rates of 92.9% in the first stent placement and without additional endoscopic procedures.

The rates of complete leakage healing after stent placement range from 70% to 85.7% in patients who develop esophageal anastomotic leakage after esophagectomy or gastrectomy [12,13,14]. When assessed by the first stent placement, the success rates slightly decrease to 51–80% [12,13,14]. Further, despite the high success rates of leakage healing, stent migration is frequent, occurring in 25–61% of the patients depending on the anastomosis type [12,13]. Various methods such as stent anchoring with clips [20], endoscopic suture fixation [21], and external fixation using thread [15] have been used to prevent stent migration. However, these did not completely prevent the migration. The stent migration rates remained to be approximately 13% for anchoring with clips method [21], 17% for suture fixation [22], and 11% for external thread fixation [13,14,15]. In the current study, the migration rate of the Niti-S Beta stent was only 7.1% (1/14 patients), and this was managed without internal or external stent fixation methods. The migration occurred in the patient with an esophago-gastrostomy anastomosis site. This low migration rate supports that the Niti-S Beta stent could be an effective treatment for anastomotic leakage after total or proximal gastrectomy with low risk of stent migration.

The thread-fix stent is frequently used for the treatment of anastomotic leakage after esophago-gastric surgery owing to its effectiveness, with low rates of stent migration [13,14]. However, it is also associated with continuous discomfort from the external fixation similar to that used in the ENBD procedure. Thus, long-term stent maintenance of four to six weeks requiring for complete leakage healing might be difficult. In this study, the duration of stent maintenance was significantly shorter in the Thread-Fix Stent Group, and thus, complete leakage healing rate by the first stent placement was significantly lower in the Thread-Fix Stent Group than in the Beta Stent Group. Additional endoscopic procedures were needed in 46.2% (6/13) of the patients. These data indicate the superiority of the Niti-S Beta stent with respect to long-term stent maintenance until leakage healing after total or proximal gastrectomy.

Outpatient management is an important advantage of the Niti-S Beta stent for anastomotic leakage treatment. Although there was no significant difference in the interval between leakage diagnosis and complete healing between the two stents, the duration of hospitalization were significantly shorter in the Beta Stent Group than in the Thread-Fix Stent Group. A total of 50% of the patients in the Beta Stent Group were on outpatient management until complete leakage healing, whereas all patients in the Thread-Fix Stent Group remained to be hospitalized because of external fixation through the nose. In addition, there was also no significant between-group difference in complications related to stent placement.

Our study has some limitations. First, this is a retrospective study conducted in a single center. Second, the patients in the Beta Stent Group and the Thread-Fix Stent Group were recruited in two different periods. Although there was no significant between-group difference in the baseline characteristics, inherent biases might not have been avoided. Third, both groups included a small number of patients. Further large-scale prospective studies are needed to validate our findings.

## 5. Conclusions

The novel Niti-S Beta stent is an effective treatment for anastomotic leakage that develops after total or proximal gastrectomy for gastric cancer, with a low rate of stent migration. It does not need external fixation, and thus has the advantage of a longer duration of stent maintenance and a shorter hospitalization than the thread-fix stent.

## Figures and Tables

**Table 1:** Baseline patient characteristics.

	Total (*n* = 27)	Beta Stent Group	Thread-Fix Stent Group	*p*
		(*n* = 14)	(*n* = 13)	
Age (years), median (IQR)	66 (59–73)	71 (60–76)	64 (59–68)	0.576
Sex, n (%)				0.29
Female	1 (3.7)	0 (0)	1 (7.7)	
Male	26 (96.3)	14 (100)	12 (92.3)	
Body mass index (kg/m^2^), median (IQR)	24.0 (22.3–26.5)	24.0 (22.3–25.9)	24.7 (23.0–26.6)	0.382
Smoking status, n (%)				0.275
Non-smoker	4 (14.8)	2 (14.3)	2 (15.3)	
Ex-smoker	18 (66.7)	11 (78.6)	7 (53.8)	
Current smoker	5 (18.5)	1 (7.1)	4 (30.8)	
Alcohol drinking, n (%)				0.883
No	10 (37.0)	5 (35.7)	5 (38.5)	
Yes	17 (63.0)	9 (64.3)	8 (61.5)	
ASA score, n (%)				0.531
1	4 (14.8)	2 (14.3)	2 (15.4)	
2	21 (77.8)	10 (71.4)	11 (84.6)	
3	2 (7.4)	2 (14.3)	0 (0)	
Gastric cancer type, n (%)				0.516
Early gastric cancer	10 (37.0)	6 (42.9)	4 (30.8)	
Advanced gastric cancer	17 (63.0)	8 (57.1)	9 (69.2)	
Type of surgery, n (%)				0.037
Total gastrectomy	23 (85.2)	10 (71.4)	13 (100)	
Proximal gastrectomy	4 (14.8)	4 (28.6)	0 (0)	
Surgical approach, n (%)				0.31
Open	16 (59.3)	7 (50.0)	9 (69.2)	
Laparoscopy	11 (40.7)	7 (50.0)	4 (30.8)	
Pathological tumor stage, n (%)				0.59
Stage I	13 (48.1)	8 (57.1)	5 (38.5)	
Stage II	5 (18.5)	3 (21.4)	2 (15.4)	
Stage III	7 (25.9)	2 (14.3)	5 (38.5)	
Stage IV	2 (7.4)	1 (7.1)	1 (7.7)	

Abbreviations: ASA, American Society of Anesthesiologists; IQR, interquartile range.

**Table 2:** Characteristics and managements of anastomotic leakages before stent placement by group.

	Beta Stent Group	Thread-Fix Stent Group	*p*
	(*n* = 14)	(*n* = 13)	
Interval between surgery and diagnosis (days), median (IQR)	8 (5–11)	7 (6–7)	0.77
Leakage site, n (%)			0.936
Esophageal anastomosis site *	12 (85.7)	11 (84.6)	
Jejunal stump	2 (14.3)	2 (15.4)	
Leakage size (mm), median (IQR)	12 (8–20)	18 (12–25)	0.091
Leakage management before stent insertion, n (%)			
Percutaneous drainage	7 (50.0)	7 (53.8)	0.842
Endoscopic procedures †	3 (21.4)	2 (15.4)	0.686
Time interval between diagnosis and stent placement (days), median (IQR)	3 (1–7)	1 (1–7)	0.676

* Esophago-jejunostomy site for total gastrectomy and proximal gastrectomy with double-tract reconstruction and esophago-gastrostomy site for proximal gastrectomy. † Endoscopic procedures include endo-clips with or without detachable snares. Abbreviations: IQR, interquartile range.

**Table 3:** Therapeutic outcomes and complications by group.

	Beta Stent Group	Thread-Fix Stent Group	*p*
	(*n* = 14)	(*n* = 13)	
Complete leakage healing on the first stent placement, n (%)	13 (92.9)	7 (53.8)	0.021
Additional endoscopic procedures, n (%)	1 (7.1)	6 (46.2)	0.021
Second stent placement	1	0	
Endoscopic clips with/without detachable snare	0	6	
Interval between stent insertion and diet initiation (days), median (IQR)			
Sips of water	3 (1–6)	4 (2–9)	0.606
Soft diet	6 (3–12)	14 (6–25)	0.118
Discharge with stent maintenance status, n (%)	7 (50.0)	0 (0)	0.003
Hospitalization after stent placement * (days), median (IQR)	16 (14–31)	32 (21–67)	0.037
Duration of stent maintenance (days), median (IQR)	28 (21–42)	18 (16–20)	0.006
Interval between leakage diagnosis and complete healing (days), median (IQR)	35 (22–48)	31 (21–54)	0.942
Complications related to stent placement, n (%)			
Erosion	7 (50.0)	5 (38.5)	0.547
Reflux symptoms	5 (35.7)	5 (38.5)	0.883
Migration	1 (7.1)	0 (0)	0.326
Stent site stricture requiring balloon dilatation	0 (0)	3 (23.1)	0.057

* Interval between stent placement and discharge. Abbreviations: IQR, interquartile range.

## Data Availability

The datasets generated during and/or analyzed during the current study are available from the corresponding author on reasonable request. The data is not publicly available due to patient privacy and the General Data Protection Regulation.
